# Correlation between renal ablation zone in contrast-enhanced CT and non-enhanced MRI during the early period following percutaneous cryoablation

**DOI:** 10.1007/s11604-022-01285-y

**Published:** 2022-05-12

**Authors:** Noriyuki Umakoshi, Toshihiro Iguchi, Takao Hiraki, Yusuke Matsui, Koji Tomita, Mayu Uka, Soichiro Kajita, Motoo Araki, Toshiharu Mitsuhashi, Hideo Gobara, Susumu Kanazawa

**Affiliations:** 1 Department of Radiology, Dentistry and Pharmaceutical Sciences, Okayama University Graduate School of Medicine, 2-5-1 Shikata-cho, Ki-taku, Okayama, 700-8558 Japan; 2 Deptartment of Radiological Technology, Okayama University Graduate School of Health Science, 2-5-1 Shikata-cho, Kita-ku, Okayama, 700-8558 Japan; 3 Department of Urology, Dentistry and Pharmaceutical Sciences, Okayama University Graduate School of Medicine, 2-5-1 Shikata-cho, Kita-ku, Okayama, 700-8558 Japan; 4 Center for Innovative Clinical Medicine, Okayama University Hospital, 2-5-1 Shikata-cho, Kita-ku, Okayama, 700-8558 Japan

**Keywords:** Kidney neoplasms, Cryosurgery, Tomography, X-Ray Computed, Magnetic resonance imaging

## Abstract

**Purpose:**

To retrospectively evaluate and correlate the contrast-enhanced computed tomography (CECT) and non-enhanced magnetic resonance imaging (MRI) during the early period following renal cryoablation.

**Materials and methods:**

Both dynamic CECT and non-enhanced MRI were performed within 4 days following cryoablation in 34 renal tumors in 33 patients. The renal volumes of the unenhanced regions on dynamic CECT (nephrogenic phase, 4 mm thickness) and the regions with signal intensity changes on non-enhanced MRI (fat-suppressed T2-weighted image, 4 mm thickness) were evaluated. Fusion images of the axial, coronal, and sagittal sections of CECT and MRI images were created from the maximum cross-section of the renal tumor, and the match score of each image was visually evaluated on a 5-point scale.

**Results:**

The mean renal volume of the unenhanced regions on CECT and those with signal intensity changes on non-enhanced MRI following cryoablation were 29.5 ± 19.9 cm^3^ (range, 4.3–97.4 cm^3^) and 30.7 ± 19.8 cm^3^ (range, 6.7–94.0 cm^3^), respectively; the difference between them was –1.17 cm^3^ (95% confidence interval [CI] –2.74, 0.40, *P* = 0.139). The Pearson’s product-moment correlation coefficient (*r* = 0.975; 95% CI, 0.951, 0.988; *P* < 0.0001) showed a strong correlation between the volumes. The average match score between CECT and non-enhanced MRI was as high as 4.5 ± 0.5 points (radiologist 1, 4.3 ± 0.5; radiologist 2, 4.7 ± 0.5). Local tumor control rate was 94.1% (32/34 tumors) and recurrence-free survival rate was 82.0% (95% CI: 64.2%, 91.5%) at 5 years.

**Conclusions:**

The region with signal intensity changes on non-enhanced MRI was strongly correlated with the unenhanced region on CECT during the early period following renal cryoablation.

## Introduction

The recent advances in and the widespread use of diagnostic imaging modalities such as ultrasound, computed tomography (CT), and magnetic resonance imaging (MRI) has facilitated the incidental detection of renal masses. The global incidence of small renal cell carcinoma (RCC) is increasing [1], for which the standard-of-care has traditionally comprised surgical resection (total or partial nephrectomy). Percutaneous ablation therapy (e.g., radiofrequency ablation, microwave ablation, and cryoablation) has recently been used to treat patients with inoperable small RCC, which has shown excellent results [2–6]. Cryoablation, in particular, is considered safe and effective as the ablation zone (also known as “ice ball”) can be confirmed in real-time during treatment. Following cryoablation, the overall survival, cause-specific survival, and progression-free survival rates at 5 years have been shown to be 84.8–97.8%, 94.3–100%, and 85.9–100%, respectively [3–6]. Furthermore, Morkos et al. have reported a 10-year cause-specific survival rate of 94% in patients with stage 1 RCC [6].

Following cryoablation, imaging evaluation using CT or MRI is indispensable for periodic follow-up, which is often complemented with the use of contrast-enhanced CT (CECT). However, CECT is contraindicated in patients with renal dysfunction and/or iodine allergy. Additionally, since cryoablation is expected to preserve renal function [7], it is often performed in patients with a decline in renal function, and it is desirable to avoid repeated CECT in such patients. In patients who are not suitable candidates for CECT, non-enhanced MRI is preferred over non-enhanced CT for evaluation of the ablation zone. This is primarily because non-enhanced MRI is capable of showing signal intensity changes in the therapeutic region [8–12]; in contrast, the therapeutic region can occasionally be unclear on non-enhanced CT images [10, 11]. However, it remains unclear whether the region with signal intensity changes on non-enhanced MRI correlates with the unenhanced region on CECT following renal cryoablation and no previous study has evaluated the correlation between them. If the ablation zone can be accurately evaluated using non-enhanced MRI, it may be beneficial for patients who cannot undergo CECT.

The purpose of this study was to retrospectively evaluate and correlate the CECT and non-enhanced MRI images that were obtained on the same day, with both modalities performed during the early period following renal cryoablation.

## Materials and methods

Our institutional review board approved this retrospective study (approval number, KEN2012-006) and waived the requirement for informed consent for the use of medical patient data. This study was conducted in accordance with the Declaration of Helsinki. Written informed consent for cryoablation and for each imaging examination was obtained from all patients.

## Inclusion and exclusion criteria

The inclusion criteria comprised: 1) patients with renal tumors treated by percutaneous cryoablation between August 2012 and June 2013 at our institution, and 2) patients with renal tumors who underwent both dynamic CECT and non-enhanced MRI on the same day within 4 days following cryoablation. The exclusion criterion comprised: patients in whom the ablation zone could not be evaluated on dynamic CECT or non-enhanced MRI following cryoablation.

## Cryoablation procedure

Cryoablation was performed percutaneously for the inpatients under CT-fluoroscopy (Aquilion 64; Canon Medical Systems, Otawara, Japan) in the interventional radiology room. All procedures were performed under conscious sedation and local anesthesia using an argon- and helium-based cryoablation system (CryoHit, Galil Medical, Yokneam, Israel) with cryoprobe (Ice-Rod or Ice-Seed, Galil Medical). The type and number of cryoprobes used and the array of cryoprobes inserted in the tumors were determined by experienced interventional radiologists based on their consensus.

## CT and MRI following cryoablation

Dynamic CECT (Discovery CT750 HD, GE Healthcare, Milwaukee, Wisconsin) and non-enhanced MRI (3 T Skyra, Siemens, Enlargen, Germany for all tumors except one [1.5 T Achieva, Philips Healthcare, Best, Netherlands]) were performed on the same day within 4 days following cryoablation to evaluate both technical success and early complications of the procedure. Dynamic CECT images were obtained before and after the intravenous administration of 300 mgI/ml contrast medium, at a dose of 2.0 g iodine per kg of weight, and a fixed injection duration of 30 s during the corticomedullary phase (36-s delay), nephrogenic phase (53-s delay), and excretory phase (240-s delay).

The obtained MRI sequences typically included axial, coronal, and sagittal sections of T2-weighted images (T2WI) both with and without fat suppression, 2D gradient-echo T1 in-phase and out-of-phase images, T2-star gradient-echo images, and diffusion-weighted images.

## Data collection

Patient background data extracted from the charts included age, sex, number of kidneys, and estimated glomerular filtration rate. Extracted data regarding lesion characteristics included maximum tumor diameter, laterality, tumor location and tumor histology. The tumor location was categorized as follows based on the definition by Gervais et al. [13] : Exophytic tumors were defined as those with a component extending into the perirenal fat but no component extending into the renal sinus fat. Parenchymal tumors were defined as those limited to the confines of the renal parenchyma, without extension into either the perirenal fat or the renal sinus fat. Central tumors were defined as those with extension into the renal sinus fat. Mixed tumors were defined as those that had components extending into both the renal sinus fat and the perirenal fat.

The dynamic CECT and non-enhanced MRI images were transferred to a workstation with SYNAPSE VINCENT^®^ ver. 5.5 (FUJIFILM Medical Co., Ltd., Tokyo, Japan) and evaluated. The detection of unenhanced regions on the dynamic CECT was the clearest during the nephrogenic phase since the most uniform enhancement is observed in the renal parenchyma. Non-enhanced MRI shows changes in signal intensity in the ablation zone following renal cryoablation, which is generally hypointense on T2WI and iso- to hyperintense on T1WI relative to the renal parenchyma [9–11]. In the renal parenchyma, the regions with signal intensity changes on non-enhanced MRI were the clearest on the T2WI or the fat-suppressed T2WI (fsT2WI) [12]. Therefore, dynamic CECT images (nephrogenic phase) and fsT2WI (T2-weighted single-shot fast spin-echo images with fat suppression [section thickness, 4 mm; gap, 0.8 mm; matrix, 320 × 256; flip angle, 120°; bandwidth, 355 Hz/px; and field-of-view, 30 cm]) were used for image evaluation.

## Evaluation of ablation zone volume and fusion images

First, the renal volumes of the unenhanced regions on dynamic CECT (nephrogenic phase, 4 mm thickness) and those with signal intensity changes on non-enhanced MRI (fsT2WI, 4 mm thickness) were evaluated. Both regions were traced on the VINCENT by two board-certified diagnostic radiologists (N.U. and T.I.), and the volume of each region was calculated. Subsequently, using VINCENT’s multi-3D application, a fusion image was created by superimposing a CECT color map image on an MRI image. Since accurate fusion images could not always be created using the automatic mode of the application, manual adjustment by the author was required in all cases. Fusion images of the axial, coronal, and sagittal sections were created from the maximum cross-section of the renal tumor, and the matching rate of each image was visually evaluated. The consistency between the unenhanced regions on CECT and those with signal intensity changes on non-enhanced MRI, including the contour of the kidney on each imaging type, was evaluated on a 5-point scale using the fusion images of the axial, coronal, and sagittal sections. The quality of alignment was rated on a scale ranging from 1 (complete lack of superimposition) to 5 (exact superimposition) (Table 1), modified according to Krishnasetty et al. [14]. Image analysis and scoring were performed independently by two board-certified diagnostic radiologists, who are also co-authors of the study, with 10 (radiologist 1, S. Kajita) and 15 years (radiologist 2, K.T.) of experience, respectively. They also calculated the minimal margins on MRI using both axial and coronal fsT2WI.

**Table 1:** Score applied for the assessment of image fusion quality

Score	Definition
1	Complete lack of superimposition of the anatomic structure
2	Difference in alignment > 10 mm
3	Difference of alignment of 5–10 mm
4	Difference of alignment < 5 mm
5	Excellent superimposition of the anatomic structure (Difference of alignment < 2 mm)

This scoring was modified according to Krishnasetty et al. [14]

## Evaluation of outcomes

The cryoblation outcomes were assessed, including the technical success, adverse events, local tumor progression, and recurrence-free survival after ablation. Technical success was defined as completion of cryoablation according to the procedure protocol [2]. Adverse events were graded according to the Common Terminology Criteria for Adverse Events (CTCAE) version 5.0. Local tumor progression was defined as the appearance of a nodular focus within or adjacent to the ablation zone [2]. Recurrence-free survival was defined as the time from the date of the cryoablation to the date of the first documented local tumor progression on image or death from any cause, whichever occurred first.

## Statistical analysis

**Fig. 1:**
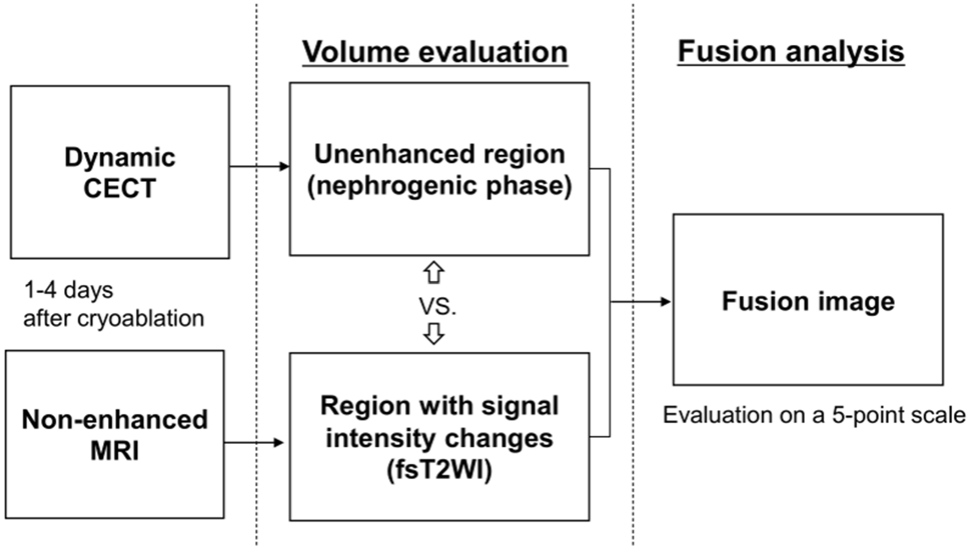
Flowchart shows how to evaluate dynamic CECT and non-enhanced MRI

Statistical analyses of the ablation zone volume evaluation and fusion image analysis were performed (Fig. 1). Values are shown as mean ± standard deviation (SD). For the ablation zone volume evaluation, statistical analysis was performed using Pearson’s product-moment correlation coefficients (r) and their 95% confidence intervals (CI). After classifying the tumors as exophytic or non-exophytic based on their location, the volume evaluation of each type was also compared. Fusion image analysis was evaluated on a 5-point scale according to a previous study [14]. The recurrence-free survival rates were estimated using Kaplan–Meier’s analysis. The analyses were performed by a statistician (T.M.) with 16 years of experience, using Stata 16.1/MP4 (Stata Corporation, College Station, TX, USA). A *P* value < 0.05 was considered statistically significant.

## Results

### Patient and tumor characteristics

Between August 2012 and June 2013, 40 renal tumors in 39 patients were treated with cryoablation at our institution. Among these, six tumors in six patients were excluded from the study. Four patients did not undergo CECT (*n* = 3) or MRI (*n* = 1). One patient had a history of multiple renal ablation treatments, and the other had a history of dialysis with reduced renal blood flow; therefore, it was difficult to evaluate the unenhanced regions on CECT in these two patients following cryoablation. Finally, 34 tumors (mean diameter, 2.2 ± 1.1 cm; range, 0.8–5.7 cm) in 33 patients (25 men and 8 women; mean age, 65.2 ± 13.9 years; range 39–87 years) were included in this study (Table 2). For the enrolled participants, dynamic CECT and non-enhanced MRI images were performed within 4 days following cryoablation (1 day, *n* = 7; 2 days, *n* = 18; 3 days, *n* = 7; and 4 days, *n* = 2).

**Table 2:** Characteristics of 33 patients and 34 renal tumors

Variable		Value
Patient characteristics (*n* = 33)		
Age (years)	Mean ± SD (range)	65.2 ± 13.9 (39–87)
Sex	Male/Female	25/8
Number of kidneys	1/2	28/5
eGFR (mL/min/1.73m^2^)	Mean ± SD (range)	66.0 ± 18.9 (32.6–127.2)
Lesion characteristics (*n* = 34)		
Size (cm)	Mean ± SD (range)	2.2 ± 1.1 (0.8–5.7)
Laterality	Right/Left	16/18
Location	Central/Parenchymal/Mixed/Exophytic	4/4/3/23
Histology	Clear cell RCC/Papillary RCC /Angiomyolipoma/Undetermined/Non-biopsy	22/1/1/3/7

*SD* standard deviation, *eGFR* estimated glomerular filtration rate, *RCC* renal cell carcinoma

Most tumors showed exophytic locations (67.6%, 23/34 tumors). Biopsy was performed on 27 tumors, most of which were histologically diagnosed as clear-cell RCC (81.5%, 22/27 tumors). Seven tumors in six patients did not undergo biopsy; however, they were clinically diagnosed as renal carcinoma. Among these, five tumors had developed in patients with hereditary diseases (von Hippel-Lindau disease [*n* = 4] or Birt-Hogg-Dubé syndrome [*n* = 1]), one was a case of renal metastasis from intrahepatic cholangiocarcinoma, and one tumor was a postoperative local recurrence of RCC.

## Ablation zone volume evaluation

**Fig. 2:**
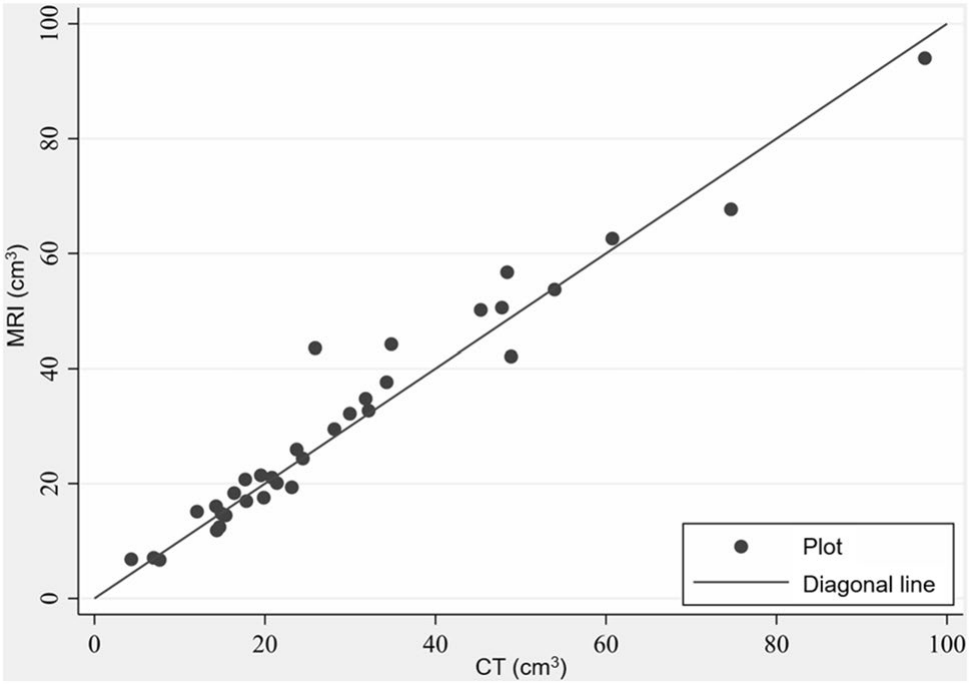
Scatter plot illustrating the linear correlation between the volume of the unenhanced regions on CECT and the regions with signal intensity changes on non-enhanced MRI

The mean renal volumes of the unenhanced regions on CECT and those with signal intensity changes on non-enhanced MRI following cryoablation were 29.5 ± 19.9 cm^3^ (range, 4.3–97.4 cm^3^) and 30.7 ± 19.8 cm^3^ (range, 6.7–94.0 cm^3^), respectively. Both values were strongly correlated with each other (*r* = 0.975; 95% CI, 0.951, 0.988; *P* < 0.0001) (Fig. 2). In 14 tumors, the average renal volume of the unenhanced regions on CECT was calculated to be 2.32 cm^3^ (range, 0.07–6.97 cm^3^) larger than the regions with signal intensity changes on non-enhanced MRI. In contrast, in 20 cases, the average renal volume of the regions with signal intensity changes on non-enhanced MRI was calculated to be 3.61 cm^3^ (range, 0.12–17.67 cm^3^) larger than the unenhanced regions on CECT. Overall, the difference between them was -1.17 cm^3^ (95% CI -2.74, 0.40, *P* = 0.139), and the renal volume of the regions with signal intensity changes on non-enhanced MRI was slightly larger than that of the unenhanced regions on CECT. A comparison of tumor locations (exophytic, 23 or non-exophytic, 11) also showed that the renal volume of the regions with signal intensity changes on non-enhanced MRI was slightly larger than that of the unenhanced regions on CECT; exophytic (–1.14 cm^3^ [95% CI: –2.80, –0.01; *P* = 0.049]), non-exophytic (–0.68 cm^3^ [95% CI: 5.12, 3.75; *P* = 0.739]).

## Fusion image analysis

**Fig. 3:**
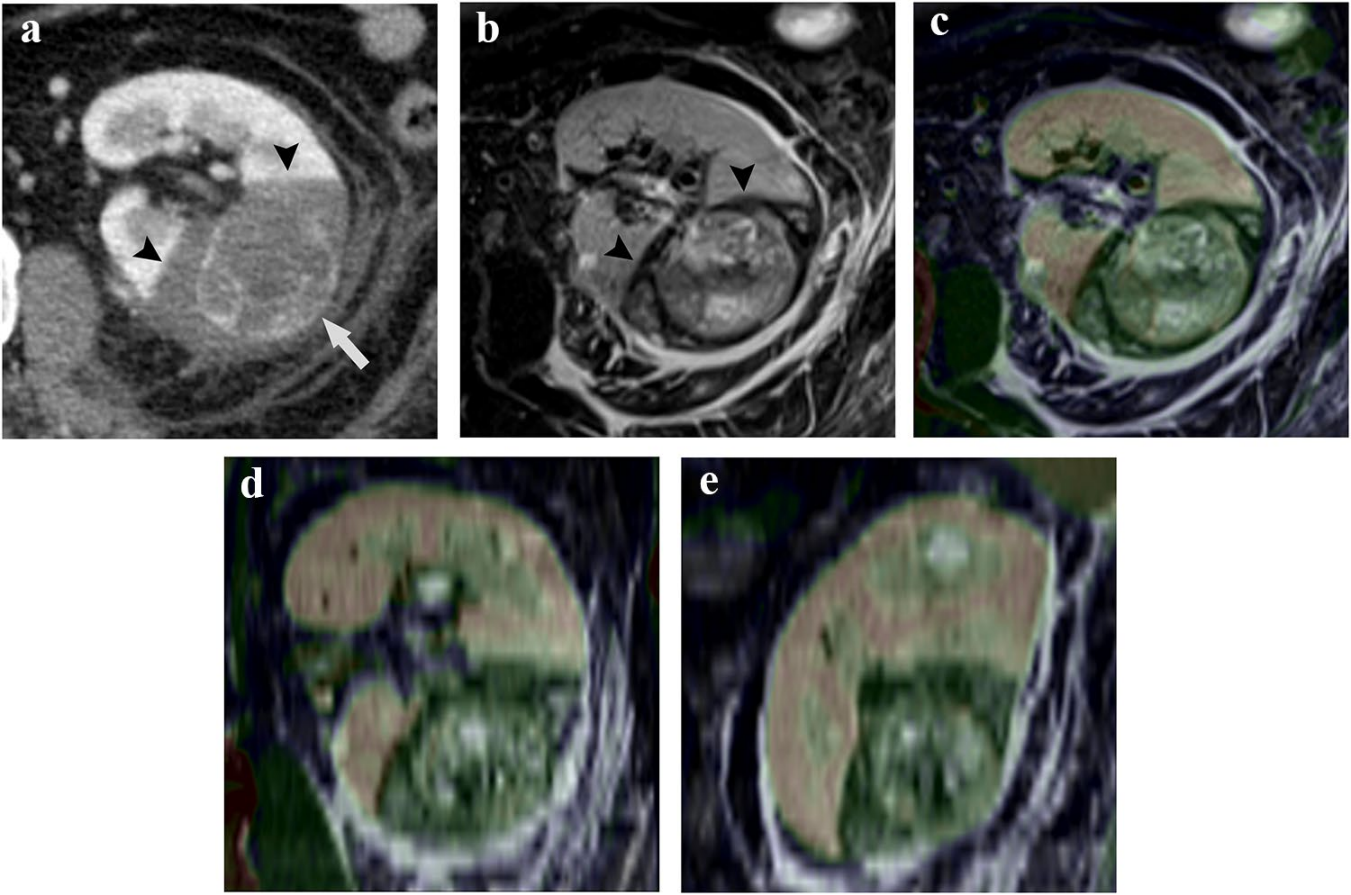
66 year-old man with biopsy-proven clear-cell renal cell carcinoma with a maximum diameters of 4.1 cm who underwent percutaneous cryoablation 2 days ago. **A** In the nephrogenic phase of dynamic CECT image, the ablation zone is visualized as the unenhanced region (arrowhead) and residual heterogeneous tumor enhancement (arrow) is shown slightly. **B** In the fat-suppressed T2-weighted MRI image, the ablation zone shows hypointensity (arrowhead) relative to the renal parenchyma. The minimal margin on MRI was 4 mm. **C, D, E,** Fusion images combining the fat-suppressed T2-weighted MRI image and the nephrogenic phase of dynamic CECT image. Axial (**C**), coronal (**D**), and sagittal (**E**) images of the maximum cross-section of the tumor. The average match score of the two board-certified diagnostic radiologists on a 5-point scale is 4.7 points (radiologist 1 [axial, 5; coronal, 4; sagittal, 4] and radiologist 2 [axial, 5; coronal, 5; sagittal, 5])

**Fig. 4:**
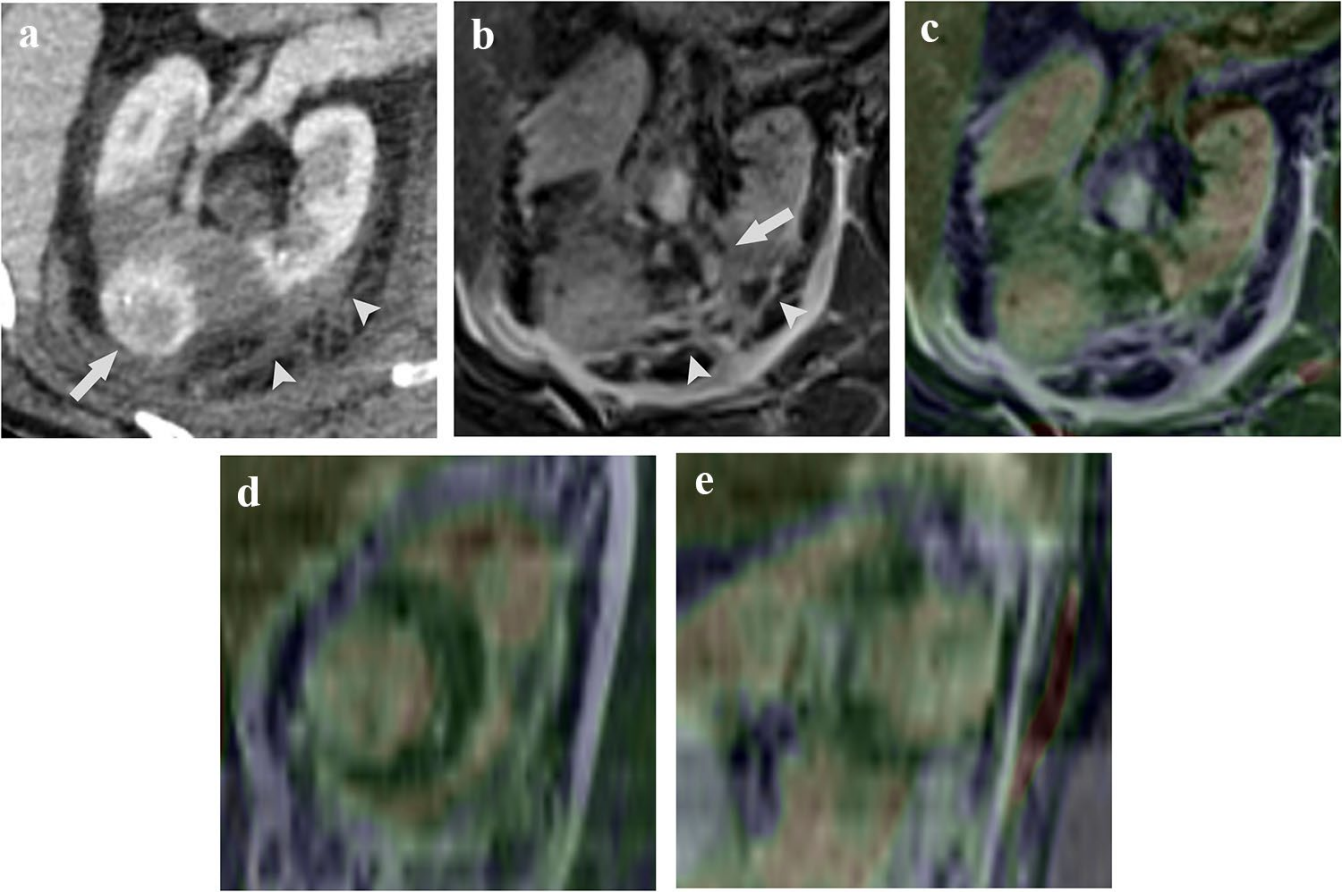
86 year-old man with biopsy-proven clear-cell renal cell carcinoma with a maximum diameters of 2.6 cm who underwent percutaneous cryoablation the day before. **A** In the nephrogenic phase of dynamic CECT image, the ablation zone is visualized as the unenhanced region with a strong appearance of perinephric changes, such as fluid collection and hematoma (arrowhead). Residual heterogeneous tumor enhancement (arrow) is shown. **B** In the fat-suppressed T2-weighted MRI image, perinephric changes such as fluid collection and hematoma show heterogeneous signal intensity changes (arrowhead), and the outline of the kidney is unclear. Although the therapeutic region shows hypointensity relative to the renal parenchyma, the left border with the renal parenchyma is slightly unclear (arrow). In this case, the renal volume of the regions with signal intensity changes on non-enhanced MRI was calculated to be 9.39 cm^3^ larger than the non-enhanced regions on CECT. The minimal margin on MRI was 4 mm. **C, D, E,** Fusion images combining the fat-suppressed T2-weighted MRI image and the nephrogenic phase of dynamic CECT image. Axial (**C**), coronal (**D**), and sagittal (**E**) images of the maximum cross-section of the tumor. The average match score of the two board-certified diagnostic radiologists on a 5-point scale is 4.3 points (radiologist 1 [axial, 4; coronal, 4; sagittal, 4] and radiologist 2 [axial, 4; coronal, 5; sagittal, 5])

The average match score of the axial, coronal, and sagittal sections of the maximum renal tumor cross-section between the dynamic CECT and non-enhanced MRI was 4.5 ± 0.5 points (radiologist 1, 4.3 ± 0.5 points; radiologist 2, 4.7 ± 0.5 points). The mean scores for the axial, coronal, and sagittal sections were 4.6 ± 0.6, 4.5 ± 0.5, and 4.5 ± 0.5, respectively. The detailed scores between the two radiologists are shown in Table 3, and there were no tumors with ≤ 2 points. A similarity was observed in the fusion images between the unenhanced regions on CECT and those with signal intensity changes on non-enhanced MRI following renal cryoablation. Representative cases are shown in Figs. 3 , 4.

**Table 3:** The match score of fusion images combining CECT image and MRI image

		Radiologist 1			Radiologist 2		
		Axial	Coronal	Sagittal	Axial	Coronal	Sagittal
Score	1	–	–	–	–	–	–
	2	–	–	–	–	–	–
	3	1	1	1	1	–	–
	4	18	20	23	8	13	10
	5	15	13	10	25	21	24

## Outcomes

All procedures were technically successful with no grade ≥ 3 adverse events. During the median follow-up period of 76 months (range, 12–110 months), two tumors showed local progression at 15 and 35 months, respectively. Among these, one tumor was treated with secondary cryoablation and the other was treated with total nephrectomy.

At the conclusion of the study, 27 patients were alive and six had died due to another disease (*n* = 5) or multiple metastases of RCC (*n* = 1). Local tumor control rate was 94.1% (32/34 tumors).Recurrence-free survival rates were 97.1% (95% CI: 80.9%, 99.6%) at 1 year, 85.3% (95% CI: 68.2%, 93.6%) at 3 years, and 82.0% (95% CI: 64.2%, 91.5%) at 5 years. The average ablation margin on non-enhanced MRI was 5.2 ± 2.3 mm (range, 0–12 mm; 0 mm, *n* = 1; 3 mm, *n* = 7; and ≥ 4 mm, *n* = 26). The ablation margins of the two tumors with local progression were 0 mm and 3 mm, respectively.

## Discussion

In this study, a strong correlation was observed in both ablation zone volume evaluation and fusion image analysis between the unenhanced regions on CECT and those with signal intensity changes on non-enhanced MRI during the early period following renal cryoablation.

In cryoablation, the target tissue is rapidly cooled to induce cell death, which results from two sequential and synergistic mechanisms. Cooling leads to the formation of intra- and extracellular ice crystals that are directly cytotoxic, leading to cell dehydration and rupture. Thawing of the frozen tissue leads to microvascular occlusion with cell hypoxia, resulting in indirect ischemic injury [15]. Consequently, the ablation zone following cryoablation can cause tissue necrosis and kill tumor cells, which has been shown to be effective in the treatment of RCC. To achieve complete tumor cell death, the target tumor must be included in the ablation zone with a margin of at least 5–7 mm, typically about 1 cm [16–18]. Although the ablated margin should be accurately assessed during and immediately following cryoablation, it may be difficult to assess it in some target areas (e.g., central RCC) using non-enhanced CT [11]. In such cases, the ablated margin may be assessed using non-enhanced MRI. Since local tumor progression was observed in only two RCCs with ≤ 3 mm margins, evaluation of margins on non- enhanced MRI during the early period may be useful as an early predictor of local tumor progression. However, it should be noted that the evaluation of margins in perirenal fat on non-enhanced MRI is difficult.

CECT is often used for post-ablation follow-up due to its advantages such as lower cost, wider availability, and easier assessment of the therapeutic region (shown as the unenhanced region) and distant metastases (e.g., lung, lymph node, and bone metastases). One of the primary aims of follow-up imaging during the early period after renal cryoablation is to detect complications such as hematoma, ureteral or collecting system injury, and bowel injury [19]. Moreover, the use of contrast medium facilitates the evaluation of active bleeding, pseudoaneurysms, and ischemic changes in surrounding organs due to cryoinjury. However, MRI usually has a limited range compared to CT, and non-enhanced MRI can only show limited information in the chest and pelvis.

On non-enhanced CT images, post-ablation changes are often unclear, although the ablation zone following cryoablation is usually observed as an area of hypo-attenuation compared to the normal renal parenchyma [10, 11]. Therefore, non-enhanced CT images may be unsuitable for evaluation of the ablation zone and ablated margins. In contrast, MRI shows changes in signal intensity in the ablation zone following renal cryoablation even without contrast enhancement, which is generally hypointense on T2WI and iso to hyperintense on T1WI relative to the renal parenchyma [9–11]. Remer et al. have reported that the ablation zone following renal cryoablation was generally iso-intense on T1WI (47/76, 61.8%) and iso- or hypointense on T2WI (72/76, 94.7%) [12].

In the 34 tumors included in this study, although the renal volume of the regions with signal intensity changes on non-enhanced MRI was slightly larger than that of the unenhanced regions on CECT, the Pearson’s product-moment correlation coefficient showed a strong correlation between the two volumes. MRI signal intensities are considered to originate from the zone of coagulation necrosis following cryoablation. In animal experiments, early histological examination following renal cryoablation showed the ablation boundary region where both necrotic and viable cells exist was to be the transition zone (TZ) [20]. In two animal studies, the width of the TZ containing mixed necrotic and viable cells at ablation boundaries was 0.70 ± 0.56 mm [20] and 0.75 ± 0.44 mm [21], respectively. The TZ shows various degrees of contrast enhancement due to the presence of viable cells on CECT and is occasionally circumscribed by an enhanced rim following liver radiofrequency ablation [22]. Since non-enhanced MRI detects signal intensity changes in the TZ more sensitively than CECT, it is possible that the region with signal intensity changes on non-enhanced MRI was calculated to be larger than the unenhanced region on CECT. However, no previous study has reported such a change in the MRI signal in the TZ. Furthermore, asymptomatic perivisceral fluid collection and hematoma, which appears as a hyperintense and hypo-intense region on T2WI, respectively, have been commonly observed during the early period following thermal ablation [10, 23]. In tumors with strong perinephric changes such as edematous change, fluid collection, and hematoma following cryoablation, the range of signal intensity change on MRI might be overestimated since the ablation zone boundary and the kidney outline were unclear on the MRI.

Since the ablation zone volume evaluation alone seemed to be insufficient as a basis for showing a correlation between the images, the fusion images were also created. FsT2WI fuses excellently with CT images; furthermore, apart from a clear delineation of the ablation zone boundary, it also depicted a clearer delineation of the border between the renal parenchyma and the surrounding adipose tissue. Therefore, fsT2WI was chosen for comparison with CECT. There are only a few reports of visual evaluation of fusion images of other organs such as the lung, heart, aortic arch, diaphragm, spine, and sternum [14, 24], and we used a modification of the fusion score evaluated by Krishnasetty et al. [14]. Independent visual evaluations by the two board-certified diagnostic radiologists showed a high concordance rate for each other’s images; however, those with strong perinephric changes tended to have lower scores. Cases in which perinephric changes such as edematous change, fluid collection, and hematoma appeared strongly following cryoablation, it was somewhat difficult to evaluate the ablation zone using non-enhanced MRI. Elucidating which cases showed strong perinephric changes following cryoablation was not within the scope of this study.

This retrospective study, with a small number of patients and tumors, has some limitations. First, all our patients (those who could undergo CECT) had normal renal function. It is unclear if the results would have been the same in patients with renal dysfunction. Second, MRI was not performed in slices thinner than 4 mm, and the CT was reconstructed to 4 mm for comparison; however, the cross-sectional images of each modality could not be perfectly matched. Further, with kidney displacement due to breathing and subtle changes in posture at the time of imaging, and with the performance of the current software, it was difficult to completely fuse images with each other. Third, since it was difficult to evaluate the entire fusion image three-dimensionally, the evaluation of the fusion image was limited to the maximum cross-section of the tumor in the axial, coronal, and sagittal sections. Fourth, the CECT and MRI image evaluation was performed within 4 days following renal cryoablation. It is uncertain if the results would have been the same during a long-term period following cryoablation.

In conclusion, the region with signal intensity changes on non-enhanced MRI was strongly correlated with the unenhanced region CECT during the early period following renal cryoablation. Non-enhanced MRI may be a preferred substitute for patients who cannot undergo CECT.
