# Correction to: Correlation between renal ablation zone in contrast‑enhanced CT and non‑enhanced MRI during the early period following percutaneous cryoablation

**DOI:** 10.1007/s11604-022-01337-3

**Published:** 2022-10-11

**Authors:** Noriyuki Umakoshi, Toshihiro Iguchi, Takao Hiraki, Yusuke Matsui, Koji Tomita, Mayu Uka, Soichiro Kajita, Motoo Araki, Toshiharu Mitsuhashi, Hideo Gobara, Susumu Kanazawa

**Affiliations:** 1 Department of Radiology, Okayama University Graduate School of Medicine, Dentistry and Pharmaceutical Sciences, 2-5-1 Shikata-cho, Ki-taku, Okayama, 700-8558 Japan; 2 Deptartment of Radiological Technology, Okayama University Graduate School of Health Science, 2-5-1 Shikata-cho, Kita-ku, Okayama, 700-8558 Japan; 3 Department of Urology, Okayama University Graduate School of Medicine, Dentistry and Pharmaceutical Sciences, 2-5-1 Shikata-cho, Kita-ku, Okayama, 700-8558 Japan; 4 Center for Innovative Clinical Medicine, Okayama University Hospital, 2-5-1 Shikata-cho, Kita-ku, Okayama, 700-8558 Japan

## Correction to: Japanese Journal of Radiology 10.1007/s11604-022-01285-y

In the original publication affiliations 1 and 3 were published incorrectly and the correct affiliations are given in this Correction.

1 Department of Radiology, Okayama University Graduate School of Medicine, Dentistry and Pharmaceutical Sciences, 2-5-1 Shikata-cho, Ki-taku, Okayama, 700–8558, Japan.

3 Department of Urology, Okayama University Graduate School of Medicine, Dentistry and Pharmaceutical Sciences, 2-5-1 Shikata-cho, Kita-ku, Okayama, 700–8558, Japan.

